# Rein Tension Signals Elicit Different Behavioral Responses When Comparing Bitted Bridle and Halter

**DOI:** 10.3389/fvets.2021.652015

**Published:** 2021-05-07

**Authors:** Marie Eisersiö, Anna Byström, Jenny Yngvesson, Paolo Baragli, Antonio Lanata, Agneta Egenvall

**Affiliations:** ^1^Department of Clinical Sciences, Faculty of Veterinary Medicine and Animal Science, Swedish University of Agricultural Sciences, Uppsala, Sweden; ^2^Department of Anatomy, Physiology and Biochemistry, Faculty of Veterinary Medicine and Animal Science, Swedish University of Agricultural Sciences, Uppsala, Sweden; ^3^Department of Animal Environment and Health, Faculty of Veterinary Medicine and Animal Science, Swedish University of Agricultural Sciences, Skara, Sweden; ^4^Department of Veterinary Sciences, University of Pisa, Pisa, Italy; ^5^Research Center “E.Piaggio”, School of Engineering, University of Pisa, Pisa, Italy; ^6^Department of Information Engineering, University of Florence, Florence, Italy

**Keywords:** negative reinforcement, horse-rider interaction, equine behavior, headstall, horse training

## Abstract

When a rider maintains contact on the reins, rein tension will vary continuously in synchronicity with the horse's gait and stride. This continuous variation makes it difficult to isolate the rein tension variations that represent a rein tension signal, complicating interpretation of rein tension data from the perspective of horse-rider interaction. This study investigated (1) the characteristics of a rein tension signal and (2) horse response to a rein tension signal for backing, comparing pressure applied by a bit (bridle), or by a noseband (halter). Twenty Warmblood horses (10 young, 10 adult) wearing a rein tension meter were trained to step back in the aisle of a stable. The handler stood next to the horse's withers, applying tension on the reins until the horse stepped back. This was repeated eight times with the bridle and eight times with the halter. Data analysis was performed using mixed linear and logistic regression models. Horses displaying behaviors other than backing showed significantly increased response latency and rein tension. Inattentive behavior was significantly more common in the halter treatment and in young horses, compared with the bridle treatment and adult horses. Evasive behaviors with the head, neck, and mouth were significantly more common in the bridle treatment than in the halter treatment and the occurrence of head/neck/mouth behaviors increased with increasing rein tension and duration of the rein tension signal. When controlling for behavior, the horses responded significantly faster and to a lighter rein tension signal in the bridle treatment than in the halter treatment. By scrutinizing data on rein tension signals in relation to horse behavior and training exercise, more can be learnt about the horse's experience of the pressures applied and the timing of the release. This can assist in developing ways to evaluate rein tension in relation to correct use of negative reinforcement.

## Introduction

Horse training commonly relies on negative reinforcement to train the horse to perform different behaviors (1). Negative reinforcement is a form of operant conditioning where the animal forms an association between its behavior and the subsequent consequences (2). The definition of negative reinforcement is that an aversive stimulus is removed upon performing the correct behavior, which increases the likelihood that the same behavior will appear again in response to the same stimulus (3). In horse training, the aversive stimulus is usually some form of pressure on the horse's body (4), which when applied acts as a signal. Pressure signals in horse training are ideally applied using light pressure first, then gradually increasing the force and/or frequency/intensity until the horse performs the correct response (5–7). However, knowledge and application of these learning principles is lacking among both professional and amateur riders (8). There is thus a need for improvements in application of negative reinforcement in horse training (9). Pressure applied via the reins, either connected to the bit in the horse's mouth or to a noseband, is commonly used for signaling to the horse to decelerate, turn, or modify its head carriage (10). The variables that comprise a rein tension signal are the magnitude and duration of rein tension and the spatial direction in which the rider applies the rein tension signal, while the release of rein tension acts as the reinforcer.

Previous rein tension studies have quantified the magnitude of rein tension in various situations, compared left and right reins, and analyzed rein tension data in relation to other variables, e.g., type of headstall, gait, and riding exercise (11), in relation to a rideability score (12), and with regard to the voluntary rein tension accepted by horses (13, 14). Several studies on rein tension have found that if the rider rides with contact on the reins, the magnitude of rein tension will largely depend on the horse's gait of travel in the order: walk < trot < canter (15–17). In an observational study of rein tension during ridden transitions, Egenvall et al. (18) documented the magnitude of rein tension one second before, during, and one second after transitions between different gaits. They found that the amount of rein tension was highly associated with the gait of travel before and after the transition, with rein tension increasing when the horse was transitioning from a slower gait to a faster gait and decreasing when the horse was transitioning from a faster gait to a slower gait (18).

Apart from any rein tension signals, tension on the reins will also vary with the movements created by the horse's gait pattern, to a large degree in trot and canter and to a lesser degree in walk (19, 20). During riding at sitting trot, rein tension has been found to fluctuate by 15–20 N on average during each stride (19). Stride-split rein tension data in the trotting unridden horse, equipped with side reins, demonstrate similar values, of ~10 N variation at a neutral rein length and 15 N variation with short reins (21). To complicate matters, the moment in the stride cycle when the rein tension peaks differs between ridden horses (during suspension phase of trot) (19) and unridden horses (during stance phase of trot) (21). The variation in rein tension magnitude is also strongly affected by the rider's handling of the reins when applying rein tension signals (22).

Due to the continuous variation in rein tension that arises when the horse is in motion, rein tension data can appear unpredictable when studying a graph of raw rein tension. It is difficult to intuitively interpret the tension variations constantly occurring throughout a riding session or assess how the horse can feel the difference between a rein tension signal and variations in tension related to the gait. Understanding the communication between horse and rider that is conveyed via the reins, while rein tension is varying continuously due to the gait and stride cycle, is a complex task. The present study was conducted in an attempt to elucidate some aspects of rein tension signals that are otherwise hidden in what appears to be a random rein tension data sequence.

Specific aims of the study were to investigate (1) the characteristics of a rein tension signal and (2) the horse's response to a rein tension signal for backing from pressure applied by a bit (bridle) or by a noseband (halter). Since the bit had a smaller contact area (~15 mm) than the halter (35 mm) and since the horse's oral structures are more sensitive than the bridge of the nose (1), the hypothesis was that backing the horse with the bridle would require lower amounts of rein tension and lead to a quicker response compared with the halter. To our knowledge, this study is the first to isolate the rein tension signal from a rein tension dataset in order to investigate the characteristics of the horse's response and the variables affecting the rein tension signal.

## Materials and Methods

The study was conducted at an equestrian center in Sweden on three consecutive days in May 2019.

### Horses

Twenty Warmblood horses were recruited for the study: 10 young (five mares, five geldings) and 10 adult horses (four mares, six geldings). The young horses were 4–5 years old (4.7 years ± 0.46) and in training under saddle for about 1 or 2 years. The adult horses were 7–15 years old (10.3 years ± 2.65) and trained in dressage and jumping for more than 4 years. All the horses were used as school horses for students studying to become riding instructors or horse trainers. The horses were either housed in single box stalls (*n* = 17) with daily turn-out into paddocks and fed forage four times per day, or were kept in a loose housing system (*n* = 3) with automatic feeding stations that provide forage about 20 times per day. The horses were healthy and met the expectations of the students' supervisors in terms of performance. Two weeks prior to data collection, all horses underwent an oral examination performed by a veterinarian specialized in equine oral health. All horses were judged fit to participate in the study. Eight of the horses participated in the study on the first day, eight on the second day, and four on the last day. Each horse was only tested on 1 day, once with the bridle and once with the halter, as detailed below.

### Rein Tension Meter

A custom-made rein tension meter was used to collect rein tension data. It consisted of a load cell (Futek, USA, weight 20 g) for each rein, wired to an amplifier and an inertial measurement unit (IMU) (NGIMU, x-io technologies, UK). The IMU had 10 bit resolution and a 3.1 V battery, and weighed 46 g. The load cells were attached by a screw to metal plates pinching the rein with screws and bolts. The amplifier-box (weight 52 g) and IMU were taped together and this package was attached to the crown piece of the bridle or halter using tape so that it was placed on the poll of the horse. The wires were attached to the sidepieces of the headstall using tape. The rein tension meter was fastened on the headstall before tacking up the horse. Rein tension data were sampled at 100 Hz and stored on a micro SD card in the IMU. The rein tension meter was calibrated on the first day of the experiment. Calibration was done by suspending 10 known weights ranging between 0 and 10 kg from each meter. This was done several times before the experiment, to confirm stability of data output.

### Experimental Set-Up

The location for the treatments was an aisle (7 m long, 2 m wide) in a building used as a grooming area at the equestrian center. On one side of the aisle there were wash racks and on the other side large metal pipes used as dividers between grooming stalls. Behind the grooming stalls, there were windows facing the stable yard. The horses could see and/or hear other horses during the entire experiment.

**Figure 1:**
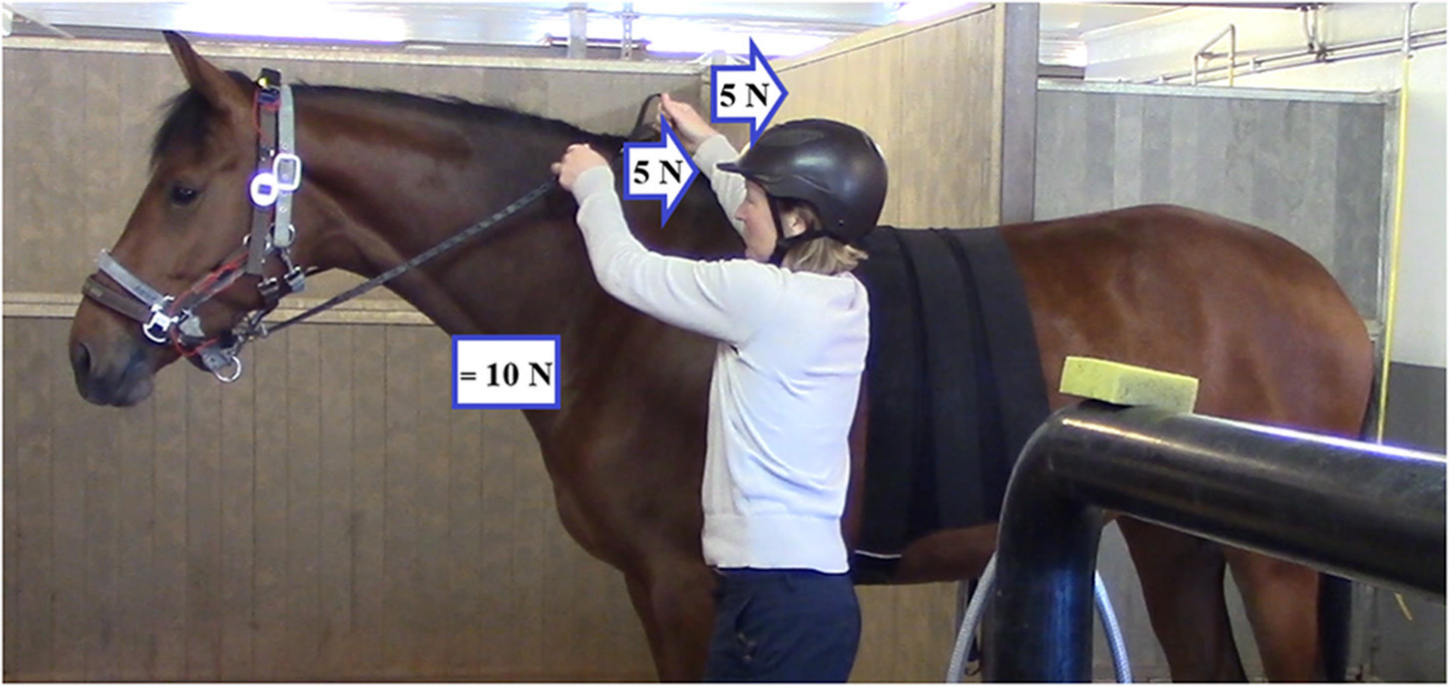
Position of the handler during the treatments. To determine the resultant force to which the horse's nose or mouth was subjected, left and right rein tension values were added. Rein tension values indicated in the figure are only examples.

The order in which the horses were tested was decided by the stable manager. Alternate horses were then assigned to one of two groups. Group 1 (four young, six adult horses) began with the bridle and Group 2 (six young, four adult horses) began with the halter. The same halter was used for all horses, but the horses wore their own bridle and bit. The noseband of the bridles was removed completely. The same reins, with the rein tension meter attached, were used for all horses throughout the experiment. All horses wore snaffle bits. There were 11 horses with three-piece snaffles, five horses with two-piece snaffles, and four horses with straight bits. The bits were between 13 and 20 mm thick closest to the rings and fitted the horses appropriately. The reins were flat leather reins (15 mm wide) with leather stoppers. The halter was full size, made of fabric, and the noseband was 35 mm wide. For the halter treatment, the reins with the rein tension meter were attached to the side rings of the halter's nose piece. For safe and easy handling during changes of headstall, all horses wore their own halter underneath the treatment headstall (Figure 1).

The handler in the experiment was author ME, who is right-handed. One horse at a time was led to one of the grooming stalls next to the aisle, where it was equipped with the headstall for the first treatment by the handler and then led to the aisle. During the entire experiment, the horses also wore an ECG monitoring system that consisted of a wide elastic girth fastened with Velcro around their belly, immediately behind the withers. The ECG data were not used in the present study.

The whole experiment was video-recorded using two video cameras. One video camera (Sony HandyCam FDR-AX53, 25 Hz) was stationary and recorded the entire trial for each horse from the frontal view, including change of tack and leading the horse between the aisle and the grooming stall. Another video camera (Canon Legria, 25 Hz) recorded the aisle, capturing a left-side view of the horse during treatment. This camera was operated by a technician following the horse's movement forwards and backwards on the aisle. The handler stood on the horse's left side during the entire experiment (Figure 1).

### Experimental Design

Each trial began by synchronizing the rein tension meter with the video recordings. For synchronization, the handler placed one hand on either side of the left rein tension meter and pulled it apart five times repeated twice, counting aloud to five. This was to produce repeated tension peaks for visual detection in each dataset. This synchronization was repeated at the end of the first treatment, and at the beginning and end of the second treatment.

The protocol for each treatment comprised the following phases:

1. Baseline—the handler stood next to the horse's withers for 2 min. The reins were resting on the horse's neck and held at the buckle by the handler, who only intervened if the horse moved or attempted something that might damage the rein tension meter.
2. Picking up the reins—the handler lifted the arms, picked up the reins, and placed the hands above the horse's withers, taking the slack out of the reins (Figure 1). In some cases, the horse's head and neck were straightened by applying tension on the right rein.
3. Rein tension signal—the handler applied tension on the reins until the horse stepped back. The handler began with light rein tension, gradually increasing the tension until the horse responded by taking a step back. If the horse stepped back immediately on a light rein tension signal in two consecutive repetitions, the criterion was raised for the next repetition, with rein tension applied until the horse stepped back an additional step. The criterion was lowered again (to fewer steps) if the horse resisted, hesitated, or seemed to have difficulty stepping back in the previous repetition. When the horse had stepped back the requested number of steps, rein tension was immediately released. If the horse took more steps than requested, this was simply ignored.
4. Rest—the horse and handler stood still on the aisle, reins held at the buckle, until 1 min had passed from the onset of the previous rein tension signal. The handler only intervened if the horse moved or attempted something that might damage the rein tension meter.
5. Repeat—points 2, 3, and 4, were repeated eight times.
6. Recovery—the horse and handler stood still on the aisle, reins held at the buckle, for a 2-min recovery period.
7. Change tack—the horse was led back to the grooming stall and tacked up with the headstall of the second treatment. Then steps 1–6 were repeated.

### Data Extraction

Using the video recordings, the video frames corresponding to the start and stop times for the different phases of the treatments were identified. During the rein tension signal, the moment when the horse lifted the first front hoof to step back was noted in the protocol as the onset of backing. The moment when the handler started lowering the hands, i.e., releasing the reins, was identified as the timing of the release. Each horse's behavior was recorded during the rein tension signal phase. Behaviors were recorded as present/absent, using the ethogram shown in Table 1.

**Table 1:** Ethogram used for behavior recording [modified after Egenvall et al. (22) and Fenner et al. (7)].

**Behavioral category**		**Behavior**	**Description**	**Horses**	***n***
Inattentive behavior	Attention	Looking at something	Directed gaze, pointed ears and immobile posture	14	35
		Investigating	Investigating the environment with nose and/or mouth	9	15
	Turning head/neck	Away	Turning the head and neck away from handler	12	33
		Toward	Turning the head and neck toward the handler for contact	9	24
Head/neck/mouth behavior	Head/neck movement	Upward	Head/neck is raised upward	17	79
		Downward	Head/neck is lowered downward	9	28
		Forward	Nose is pushed forwards	11	22
		Backward	Nose is drawn in toward the chest	6	11
		Toss	Quick upward vertical movement of the head	8	14
	Mouth behavior	Biting on bit	The bit is pulled up inside the mouth and horse is biting on it	12	28
		Open mouth	Visible gap between upper and lower jaw	17	58

*For head/neck movements, behaviors were recorded when the horse moved their head and neck in any of the directions described. Horses is the number of horses showing each behavior and n is the number of rein tension signals when each behavior was present*.

### Data Analysis

Rein tension data and video recording protocols for each trial, including start and stop times for the different phases and the behavioral recordings, were imported into Matlab (MathWorks Inc., MA, USA) and analyzed using custom-made code. Since the peak rein tension acted as the aversive stimulus, maximum rein tension was used in data analysis. Maximum rein tension was determined for the left and right reins during the phases picking up the reins and rein tension signal for descriptive statistics. The sum of the left and right maximum rein tension was computed for the rein tension signal. Response latency (time to response) was defined as the time between onset of the rein tension signal until the onset of backing as defined above. The time from onset of backing until timing of the release was calculated for repetitions of one step back. Behaviors were partitioned into two categories: Head/neck/mouth behavior (containing all head/neck movement and mouth behavior) and inattentive behavior (including attention and turning head/neck behavior) (see ethogram in Table 1).

### Statistical Analysis

A dataset with discrete rein tension values calculated per rein and phase, duration of the different phases, and behavioral records was imported into R (version 1.2.5019, RStudio, MA, USA). Descriptive statistics (R packages: tidyverse, ggplot2, dplyr, gapminder) were calculated for the phases picking up the reins and rein tension signal. The main statistical analysis was done using linear mixed and logistic mixed regression models (R packages: lmerTest, lme4, emmeans). The outcome variables maximum rein tension during the rein tension signal and response latency were modeled using linear mixed model. These variables were not normally distributed and were transformed along the ladder of powers to find the most suitable transformation, which was deemed to be square root transformation. Normality after transformation was checked by plotting Pearson's residuals. The explanatory variables were: Headstall (bridle/halter), age group (young/adult), number of steps (1–3), and 11 dichotomous behavior variables (looking at something, investigating, turning away, turning toward handler, head upward, head downward, head forward, head backward, head toss, biting on the bit, and open mouth), all entered as class variables. Horse and the interaction between horse and treatment group (order of treatment) were modeled as random variables. The full model was first tested including three-way-interactions between the three design variables (headstall, age group, and number of steps). Backwards reduction was done manually, while the three design variables were forced into the final models (without interactions). Akaike's information criterion (AIC) was evaluated during modeling. Bonferroni correction was used while reducing the model. Estimated marginal means were calculated for all variables and contrast *p-*values were used to determine significant differences between level combinations. Contrasts of more than two levels were Tukey-adjusted for multiple comparisons. *P* < 0.05 were considered significant. The covariance structure was set to unstructured.

In addition, logistic regression models were made with the behavioral category variables head/neck/mouth behavior and inattentive behavior as outcome. Headstall and age group were explanatory variables in the inattentive behavior model, while headstall, maximum rein tension, and response latency were explanatory variables in the head/neck/mouth behavior model. Maximum rein tension and response latency were tested for linearity by modeling these variables as categorical variables in the form of equidistant categories and confirming consistent increments between each pair of categories. Horse was included as a random variable.

## Results

For each of the 20 horses, eight rein tension signals were applied with the bridle and eight rein tension signals with the halter, resulting in 320 rein tension signals in total, i.e., 160 rein tension signals with the bridle and 160 rein tension signals with the halter. All horses completed the experiment and all data were retrieved for analysis.

### Rein Tension Signal and Horse Response

**Figure 2:**
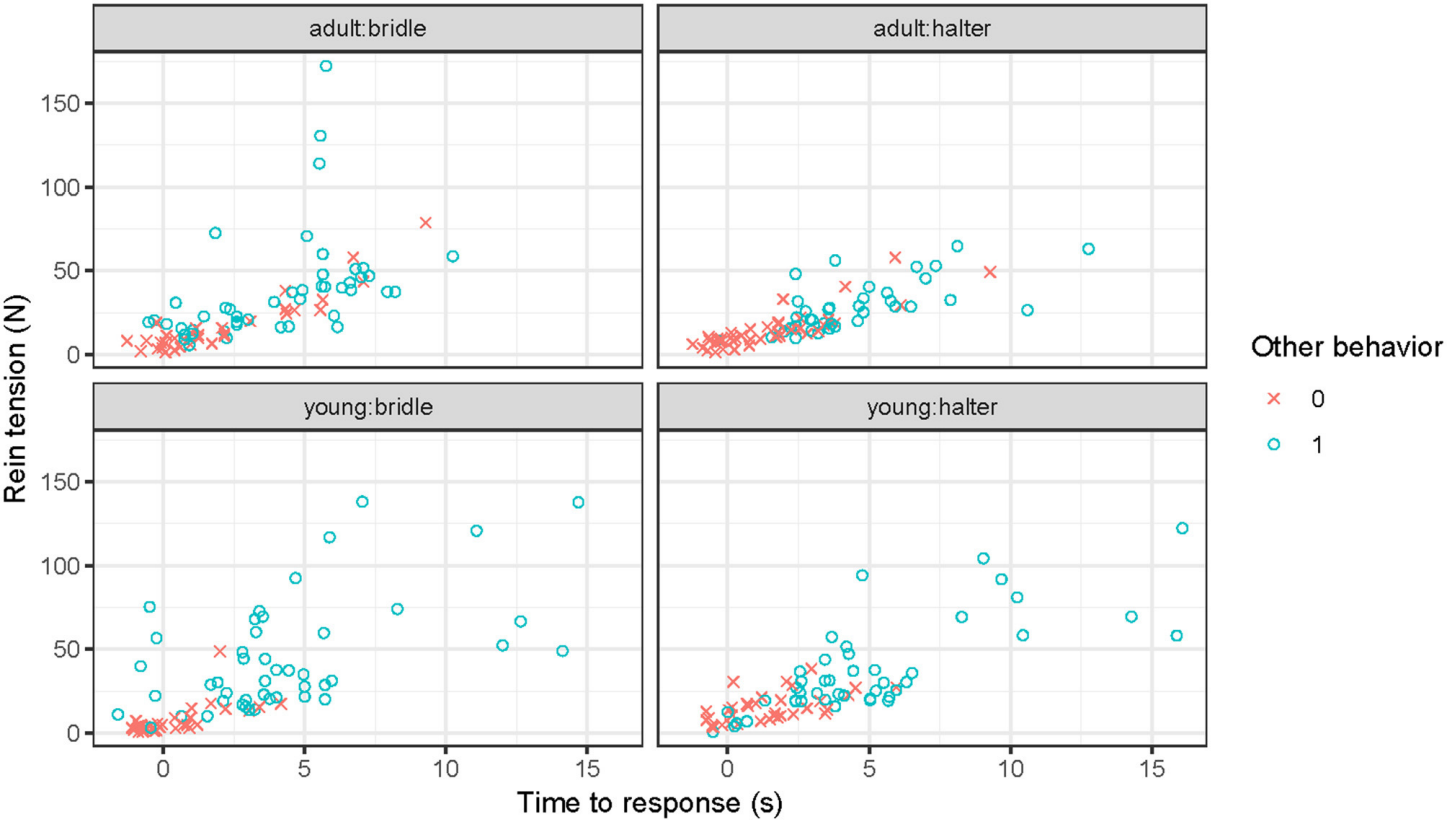
Response latency plotted against maximum rein tension (sum of left and right rein) for bridle and halter for young and adult horses. Color and shape by presence/absence of behaviors other than backing. Data include 20 horses, backing in response to a rein tension signal, each performing eight repetitions with a bridle and eight repetitions with a halter, yielding 320 rein tension signals in total. Each dot represents one of the rein tension signals for one horse. Note that the x-axis starts before zero, as some horses responded already while the handler was picking up the reins. Behaviors included are: Looking at something, investigating, turning away, turning toward the handler, head upward, head downward, head forward, head backward, head toss, biting on the bit, and open mouth.

**Figure 3:**
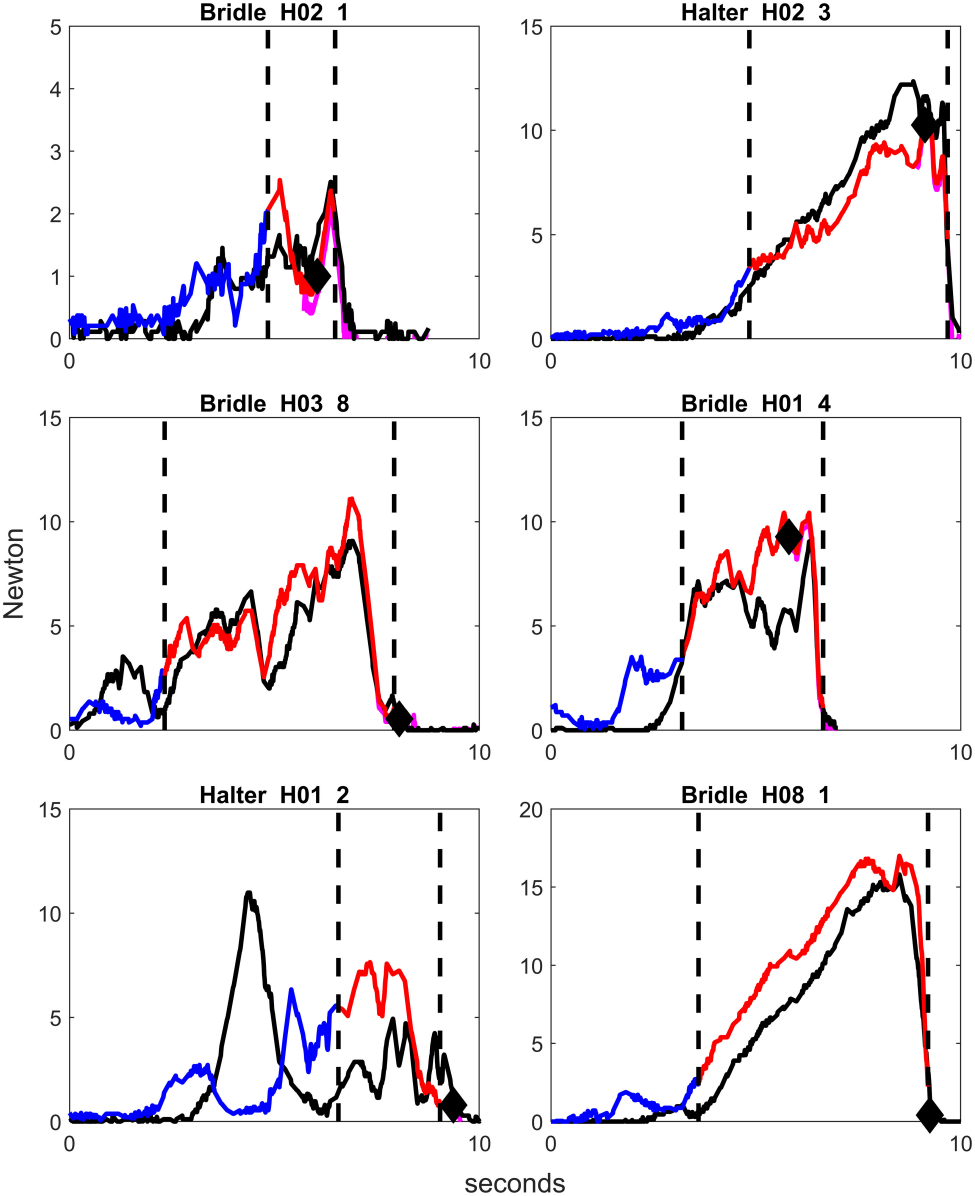
Examples of graphs of rein tension signals using bridle or halter in different horses, from repetitions where the handler applied rein tension for one step back and with no other behavioral response than backing. Blue color on the rein tension curve illustrates the phase picking up the reins, red color illustrates the rein tension signal, and purple illustrates the horse stepping back. Only the right rein is colored. The first vertical dotted line demonstrates the onset of the rein tension signal and the second vertical dotted line displays the lowering of the handler's hands, i.e., release of the rein tension signal. The black diamond signifies the onset of backing. H = Horse, first number is horse number, second number is repetition number. Note that the scale on the y-axis differs slightly between graphs.

**Figure 4:**
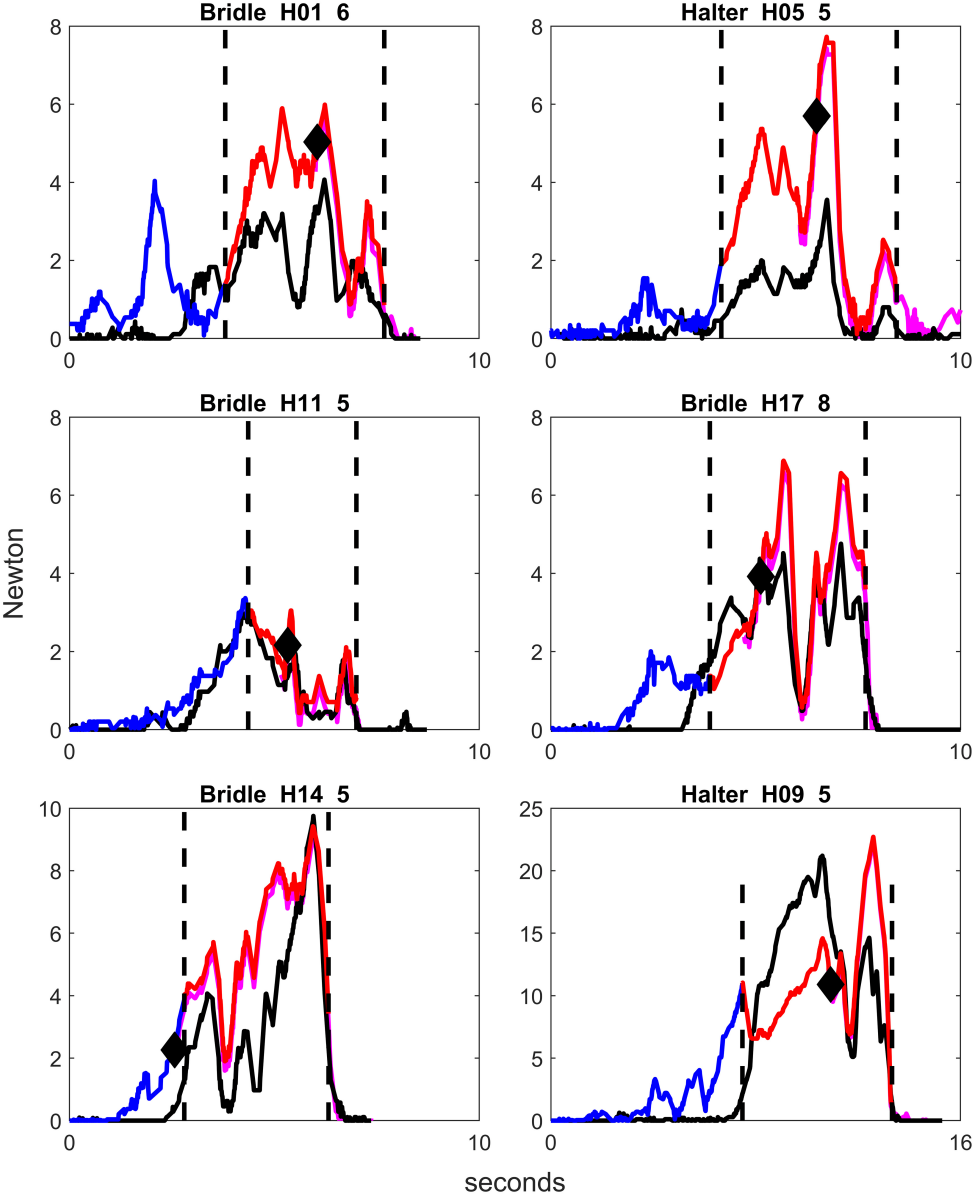
Examples of graphs of rein tension signals using bridle or halter in different horses, from repetitions where the handler applied rein tension for two steps and with no other behavioral response than backing. Blue color on the rein tension curve illustrates the phase picking up the reins, red color illustrates the rein tension signal, and purple illustrates the horse stepping back. Only the right rein is colored. The first vertical dotted line demonstrates the onset of the rein tension signal and the second vertical dotted line displays the lowering of the handler's hands, i.e., release of the rein tension signal. The black diamond signifies the onset of backing. The decrease in magnitude of rein tension shown after the onset of backing demonstrates the release of rein tension for the first step back. H = Horse, first number is horse number, second number is repetition number. Note that the scale on both the x-axis and the y-axis differs slightly between graphs.

**Figure 5:**
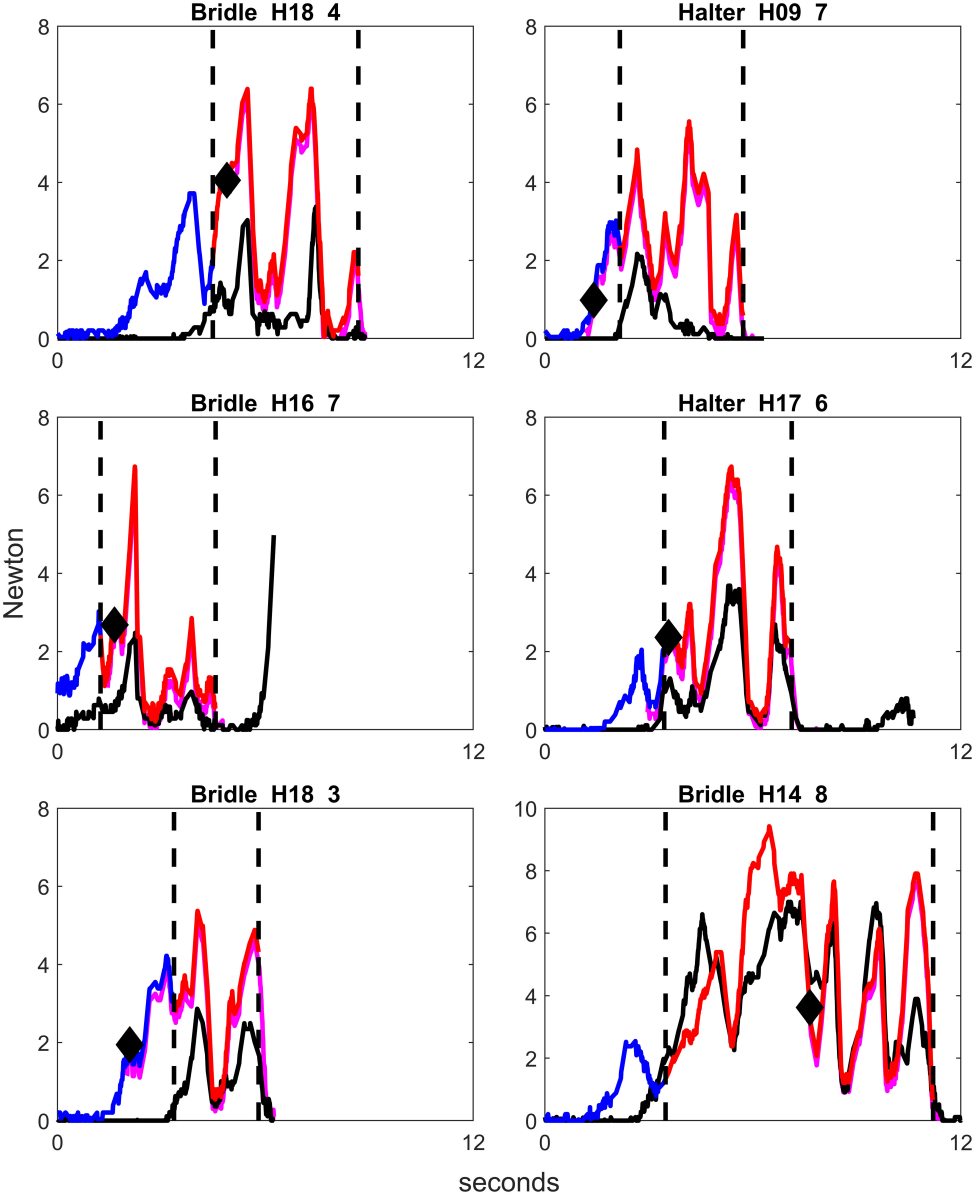
Examples of graphs of rein tension signals using bridle or halter in different horses, from repetitions where the handler applied rein tension for three steps back and with no other behavioral response than backing. Blue color on the rein tension curve illustrates the phase picking up the reins, red color illustrates the rein tension signal, and purple illustrates the horse stepping back. Only the right rein is colored. The first vertical dotted line demonstrates the onset of the rein tension signal and the second vertical dotted line displays the lowering of the handler's hands, i.e., release of the rein tension signal. The black diamond signifies the onset of backing. The repeated decreases in magnitude of rein tension after the onset of backing demonstrate the release of rein tension accompanying each step back. H = Horse, first number is horse number, second number is repetition number. Note that the y-axis differs slightly between graphs.

The response latency (time to response) and the magnitude of rein tension (sum of left and right rein) increased simultaneously, with the relationship estimated to rho 0.69 [Pearson's product-moment correlation, CI [0.63, 0.74], *p* < 0.001]. This relationship, divided by age group and headstall, is illustrated in Figure 2. Figures 3–5 show examples of raw rein tension signals and the diversity in appearance of these rein tension signals, comparing number of steps and different horses.

In 18% of the rein tension signals, the horse responded before the rein tension signal was applied, i.e., started to step back during the phase picking up the reins (22% for bridle, spread over 15 horses; 14% for halter, 10 horses). When the horse started backing before the rein tension signal was applied the response latency variable became negative, as can be seen in Figure 2. The release of rein tension was given within one second from onset of backing in 91% of the rein tension signals for bridle and 92% for halter (median 0.5 s bridle/halter).

The correlation between maximum rein tension for left and right rein was estimated to rho 0.86 [Pearson's product-moment correlation, CI [0.83, 0.89], *p* < 0.001]. Descriptive statistics for picking up the reins and the rein tension signal phases can be found in Table 2.

**Table 2:** Descriptive statistics on time variables and maximum rein tension of the phases picking up the reins and rein tension signal.

			**Time (s)**			**Maximum rein tension (N)**				
	**Headstall**	**Number of steps**	**Time to response**	**Duration**	**Rein (left/right)**	**Mean**	**SD**	**Median**	**Range**	**IQR**
Picking up the reins	Bridle (160)	n/a	n/a	3.8	L	4	7	2	0–54	1–4
		n/a	n/a		R	6	10	4	0.9–112	2–5
	Halter (160)	n/a	n/a	4.1	L	4	6	2	0–49	1–3
		n/a	n/a		R	5	5	3	0.7–31	2–5
Rein tension signal	Bridle	1 (105)	3.9	4.5	L	16	16	11	0–75	5–21
					R	19	18	15	0.9–97	7–24
		2 (37)	1	3.8	L	8	10	6	0–38	3–9
					R	9	9	7	0.5–40	4–10
		3 (18)	0.6	4.3	L	8	9	3	1.5–29	2–10
					R	10	11	6	2–41	5–9
	Halter	1 (89)	4.3	4.8	L	15	13	12	0–60	6–17
					R	16	13	13	0.8–70	8–21
		2 (53)	1.9	4.4	L	8	5	7	0.2–21	4–9
					R	9	6	8	2–39	6–10
		3 (18)	0.8	4.7	L	6	5	5	0–17	3–9
					R	9	4	7	2–18	6–13

*Numbers in brackets are number of rein tension signals. Data include 20 horses each performing eight repetitions with the bridle and eight repetitions with the halter, resulting in 320 rein tension signals. Rein tension was applied with light tension first and then increased until the horse responded by stepping back*.

### Variables Affecting Rein Tension Signal and Horse Response

The results from the final, reduced model of response latency (Tables 3, 4), revealed that, when controlling for behavior, the horses responded significantly faster in the bridle treatment (~0.5 s, Table 3) than in the halter treatment. Presence of the behaviors looking at something, investigating, turning toward handler, head upward, head forward, and open mouth was always associated with a significantly longer response latency than when absent (*p* < 0.01). The behavior investigating increased time the most, followed by looking at something, turning toward handler, and head forward (~2 s; Table 3). Two and three steps back were associated with significantly shorter response latency compared with one step back. The same model with behavior excluded resulted in no significant differences between the bridle and halter treatments, while the difference for two and three steps back remained significant (see Supplementary Material).

**Table 3:** Contrasts between variables within each category for the response latency model and the rein tension model.

**Model**	**Category**	**Variable**	**Estimate**	**SE**	**Lower CI**	**Upper CI**	***p*-value**
Response latency	Headstall	Bridle-halter	−0.62	0.24	−1.11	−0.14	0.013
	Age group	Adult–young	0.48	0.38	−0.31	1.26	0.220
	Number of steps	1–2	1.27	0.24	0.68	1.86	<0.001
		1–3	1.73	0.31	0.97	2.49	<0.001
		2–3	0.46	0.30	−0.27	1.18	0.290
	Behavior	Looking at something	2.16	0.40	1.34	2.97	<0.001
		Investigating	2.66	0.63	1.37	3.95	<0.001
		Turning toward handler	2.09	0.50	1.05	3.12	<0.001
		Head upward	1.34	0.28	0.78	1.91	<0.001
		Head forward	1.82	0.52	0.76	2.88	0.002
		Open mouth	1.05	0.36	0.32	1.79	0.007
Rein tension	Headstall	Bridle-halter	−4.88	1.64	−8.19	−1.58	0.005
	Age group	Adult-young	2.62	2.11	−1.74	6.98	0.226
	Number of steps	1–2	3.35	1.33	0.10	6.60	0.042
		1–3	3.32	1.84	−1.18	7.81	0.184
		2–3	−0.03	1.83	−4.45	4.38	1.000
	Behavior	Looking at something	9.02	2.3	4.34	13.70	<0.001
		Investigating	22.40	4.41	13.40	31.40	<0.001
		Turning away	6.71	2.24	2.14	11.30	0.005
		Head upward	4.72	1.57	1.52	7.93	0.005
		Head downward	19.40	4.02	11.20	27.60	<0.001
		Head forward	23.40	4.4	14.40	32.30	<0.001
		Open mouth	12.50	2.61	7.20	17.80	<0.001

*Estimate is the estimated difference in response latency (s) and rein tension (N) between levels within the variables headstall, age group, number of steps, and presence/absence for each behavior, where presence is when only the listed behavior is present and absence is when no behavior is present. A positive value indicates longer latency or higher rein tension for the first alternative listed within each variable. For behavior, the contrasts show how much longer time (s) and higher rein tension (N) were when each behavior was present compared with absent. The variable number of steps is Tukey-adjusted for multiple comparisons. Each model includes the response to a rein tension signal by 20 horses, making eight repetitions of backing in response to a rein tension signal wearing a bridle and eight repetitions wearing a halter. R code and output can be found in Supplementary Material*.

**Table 4:** Back-transformed least square means from the response latency model (s) and the rein tension model (N) for headstall, age group, and number of steps when controlling for behavior, and presence/absence for behavior.

**Model**	**Category**	**Variable**	**Estimate**	**SE**	**Lower CI**	**Upper CI**
Response latency	Headstall	Bridle	0.59	0.24	0.13	1.09
		Halter	1.21	0.25	0.72	1.73
	Age group	Adult	1.13	0.30	0.55	1.77
		Young	0.65	0.27	0.12	1.25
	Number of steps	1	1.93	0.27	1.41	2.49
		2	0.66	0.24	0.20	1.17
		3	0.20	0.29	−0.34	0.82
	Behavior	Looking at something	3.04	0.46	2.17	4.00
		Investigating	3.55	0.68	2.29	4.96
		Turning toward handler	2.97	0.56	1.93	4.14
		Head upward	2.23	0.34	1.57	2.94
		Head forward	2.71	0.58	1.64	3.91
		Open mouth	1.94	0.40	1.18	2.78
Rein tension	Headstall	Bridle	9.59	1.35	7.07	12.50
		Halter	14.47	1.56	11.50	17.80
	Age group	Adult	13.30	1.68	10.00	17.00
		Young	10.60	1.53	7.71	14.00
	Number of steps	1	14.20	1.40	11.49	17.10
		2	10.80	1.36	8.27	13.70
		3	10.90	1.83	7.53	14.80
	Behavior	Looking at something	20.90	2.65	16.01	26.50
		Investigating	34.30	4.70	25.68	44.20
		Turning away	18.60	2.55	13.92	24.00
		Head upward	16.60	1.93	13.01	20.70
		Head downward	31.30	4.34	23.34	40.50
		Head forward	35.30	4.79	26.47	45.30
		Open mouth	24.40	2.91	19.02	30.50

*For headstall, age group, and number of steps, the column estimate shows the estimated response latency and rein tension when behavior was controlled for, i.e., none of the behaviors was present during the rein tension signal. For behavior, the estimate value shows the estimated response latency and rein tension when each behavior was present during the rein tension signal. Each model included the response to a rein tension signal by 20 horses, making eight repetitions of backing in response to a rein tension signal wearing a bridle and eight repetitions wearing a halter. R code and output can be found in Supplementary Material*.

In the model with maximum rein tension as the outcome, rein tension was significantly lower in the bridle treatment compared with the halter treatment (~5 N; Table 3), when controlling for behavior, i.e., if no other behavior was present. All behaviors except biting on the bit, turning toward the handler, head toss, and head backward were associated with significantly more rein tension when present compared with when absent (*p* < 0.01). In particular, investigating, head forward, and head downward increased rein tension most when present compared with when absent (~20 N; Table 3). Two steps back was associated with significantly lower rein tension than one step back. The same model with behavior excluded revealed no significant differences between the bridle and the halter treatment, but two steps back remained significant (see Supplementary Material).

The odds of head/neck/mouth behavior being displayed increased significantly with increasing rein tension, OR 1.048 (95% CI 1.02, 1.08), and response latency, OR 1.34 (95% CI 1.13, 1.62). The odds of the horse showing head/neck/mouth behavior thus increased with 5% for every added newton rein tension and with 34% for every added second the rein tension signal was applied (see Supplementary Materials for calculations). There was significantly less head/neck/mouth behavior during the halter treatment, OR 0.22 (95% CI 0.12, 0.42), compared with the bridle treatment. In the halter treatment, there was significantly more inattentive behavior compared with in the bridle treatment, OR 1.76 (95% CI 1.03, 3.05), and the young horses performed these behaviors significantly more often than the adult horses, OR 4.42 (95% CI 1.73, 13.41).

## Discussion

Overall, the results showed that the horses responded significantly faster and to a lighter rein tension signal in the bridle treatment compared with the halter treatment, when controlling for behavior, confirming the starting hypothesis. However, the bridle treatment was associated with significantly more head/neck/mouth behaviors than the halter treatment, and the occurrence of head/neck/mouth behavior increased with increasing magnitude and duration of the rein tension signal. All behaviors in the head/neck/mouth category could be classified as evasive or resistance behaviors aimed at either escaping or resisting the pressure applied (Table 1). These results suggest that the bridle was perceived as more aversive by the horses, since they showed more of these evasive and resistance behaviors during the rein tension signals applied with the bitted bridle compared with the halter. Horses may also associate different equipment with different activities. Inattentive behaviors were significantly more common in the halter treatment and in young horses, indicating that young horses in particular may have associated the halter with non-training time and thus their attention was more on other things than on paying attention to rein tension signals.

Interestingly, there was no significant difference between young and adult horses in magnitude of rein tension or response latency. There was, however, a tendency for the young horses to respond faster and to a lighter rein tension signal (see estimates in Table 4), indicating that perhaps the adult horses had habituated to the rein tension signal to some extent and were thus less responsive. Nevertheless, habituation to the bit is not necessarily a consequence of having more years being trained with a bit, but is rather a consequence of how the horse's training has been conducted. The horses in our study were all school horses and were thus teaching riders to refine their skills in equitation on a daily basis. It is likely that their different riders have diverse skills in always beginning with a light rein tension signal (before gradually increasing) and releasing rein tension promptly, both of which are important skills to maintain lightness and avoid habituation to rein tension (23). By scrutinizing data on rein tension signals in relation to horse behavior and ridden exercise, more can be learnt about the horse's experience of the pressures applied and riders' timing of the release. This can assist in developing ways to evaluate rein tension in relation to the correct use of negative reinforcement and can ultimately increase the welfare of horses during training.

When a horse responded immediately to a light rein tension signal during the course of the experiment, the handler raised the criterion and applied rein tension for an additional step. Accordingly, rein tension applied for two or three steps had a shorter response latency and rein tension was lowest when the horse responded already during picking up the reins. This suggests that a quick response to a rein tension signal is crucial for keeping rein tension at a minimum (Figure 2). By focusing on making sure that the horse understands each rein tension signal, rein tension is more likely to be kept at low levels during a training session (23). The mean maximum amount of rein tension during the picking up the reins phase was ~9 N (sum of left and right rein) and in 18% of the rein tension signals the horse was backing already during picking up the reins. This amount of rein tension, or less, was thus enough to elicit a response.

All behaviors recorded except biting on the bit, head toss, and head backward were associated with a significant increase in rein tension and/or response latency when present compared with when absent (Figure 2). The behavior investigating, which increased both magnitude of rein tension and response latency, implied that the horse's attention was on investigating the environment, rather than responding to the rein tension signal. The behaviors head forward and head downward significantly increased rein tension, since the horse moved its head in the opposite direction as the handler applied the rein tension signal. These behaviors were also recorded by Egenvall et al. (22) during riding when the release of rein tension was withheld until a complete correct response was obtained. It is thus likely that the horses in the present study moved their head forward/downward in an attempt to alleviate the pressure from the bit, as doing this forcefully could pull the reins out of the hands of a rider. This behavior was likely not performed for the first time in this study, but rather previously learnt by repeated success in getting relief from rein tension by the horse pushing their head and neck in the opposite direction. In this experiment, all behaviors recorded during the rein tension signal phase likely reflect the horse's understanding of what can lead to release of rein tension, and/or its motivation/eagerness to get relief from rein tension.

The type of halter used in this study is generally used for leading and grooming, and not primarily for riding horses. We chose the halter to compare with the bitted bridle because we wanted a headstall that would be as comfortable as possible for the horse, allowing us to compare the bit with no bit, rather than with other aversive equipment. The soft textile noseband of the halter used in this study likely created a more comfortable pressure on the horse compared with the pressure from the bit in the mouth, as the force was applied on a larger contact area (noseband of halter 35 mm, bit 13–20 mm in width), thus yielding a lower pressure, and on a less sensitive body part (the bridge of the nose compared with the mouth). However, it is difficult to estimate the pressure applied to these two anatomical regions, as both are irregularly shaped and consequently small pressure points are created (24). In other words, bitless headstalls in general are not necessarily more comfortable for horses.

The reason for choosing backing from a standstill, by the unridden horse, was to reduce the number of variables that could lead to fluctuations in rein tension, i.e., gait, stride, and/or (un)steadiness of the rider's hands/handling of the reins. This approach was intended to isolate the actual rein tension signal, in order to learn more about the appearance and features of rein tension signals, knowledge that could later be applied to study rein tension signals during movement.

The plots of rein tension signals revealed that the peaks of rein tension were distributed along the time axis (x-axis), while the magnitude and frequency of the peaks (y-axis) differed between the horses (Figures 3–5). It should be noted that all signals were given by the same handler in a consistent position. There were a few common features in all rein tension signals. The most obvious was that rein tension increased gradually during the rein tension signal, but returned to zero the moment that rein tension signal was released. During repetitions where the rein tension signal was applied for two or three steps back, a reduction in rein tension magnitude accompanied each step (Figures 4, 5).

During data collection in this study, lifting of the first front hoof to step back was the feedback to the handler that the horse had responded to the rein tension signal. The timing of the release was thus coupled with lifting of the front hoof, i.e., the onset of backing. It is often stated that the release of pressure/tension has to be given immediately once the horse gives the correct response (5–7). How “immediate” is defined in this context is not clear, but we believe that releasing rein tension within one second of the horse's response would be immediate enough for the horse to make an association between its behavior (backing) and the following consequences (relief from pressure on the mouth/nose).

The results in this study show the rein tension signals given by one single handler, and thus reflect between-horse variation in response to a particular rein tension signal, rather than the traits of rein tension signals in general. It would be interesting to investigate further the variables that influence understanding and motivation among horses to respond to rein tension signals and the variation in application of rein tension signals among riders. Future studies should focus on developing methods to identify rein tension signals in a rein tension dataset of horse-rider interaction in movement.

The position of the handler is another factor to consider when interpreting the results from this study. Tension on the right rein was consistently slightly higher than on the left rein (Table 2), most likely due to the handler's position on the horse's left side. The horse was more inclined to turn toward the handler and the more open area on their left side, so the handler had to direct the horse's head straight from time to time using the right rein, thus increasing tension primarily on the right rein. Another issue to consider when interpreting the results from this study is that all the horses were from the same equestrian center, and thus trained under similar conditions and following similar procedures and training ideologies.

In rein tension studies, it is common to present the magnitude of rein tension as either a mean value for the left and right rein, or as separate values for the left and right rein. However, it is important to remember that when two forces are pulling on an object in the same direction, these forces should be added to show the resultant force. If, e.g., there is 5 N on the left rein and 5 N on the right rein, the resultant tension that the horse is experiencing as pressure applied is 10 N (Figure 1). In this study, we decided to use the resultant force for our computations and the reader should take this into consideration when comparing the magnitude of rein tension in this and other rein tension studies.

It should be borne in mind that when escalating pressure signals are used as a means of communication, there is always a risk of causing the horse discomfort, pain, and even physical injury (25). Bridles with bits and bitless alternatives both press on sensitive structures of the horse's head and mouth when rein tension is applied (26). Further, mouth injuries connected to use of bridles are common in horses (27, 28). Scrutinizing the characteristics of rein tension signals may thus yield clues to improving horse welfare during training and riding, ultimately increasing awareness of signals and how the horse perceives these. It is likely that horses would benefit from riders learning to use negative reinforcement in a more sophisticated way, e.g., by reducing the magnitude of rein tension signals, being more prompt in releasing rein tension, and recognizing how little rein tension is actually needed to elicit a response.

## Conclusions

This study of rein tension in unridden horses at a standstill had the advantages of removing variables such as gait, stride, and the rider's influence, and provided a rein tension dataset that was used to scrutinize rein tension signals. Quantification of rein tension signals and the horse's response revealed a wide range in both magnitude of rein tension and response latency (time to response) between the horses. The most prominent finding was that horse behavior during the rein tension signal was significantly associated with both magnitude of rein tension and response latency. Horses that had their attention on other things or moved their head forward and/or downward during the rein tension signal had the greatest magnitude of rein tension and the longest response latency. Likewise, occurrence of head/neck/mouth behavior increased with increasing duration and magnitude of rein tension, and the bridle treatment was associated with significantly more head/neck/mouth behaviors than the halter treatment. The horses that responded quickly to the rein tension signal had the lowest rein tension. In future studies of rein tension signals, we suggest measuring three key variables: response latency, timing of release of the rein tension signal, and behavior of the horse during the rein tension signal. In particular, the horse's behavior needs to be considered when interpreting rein tension data, as the numerous behaviors a horse can perform will affect the magnitude of rein tension. Scrutinizing data on rein tension signals in relation to horse behavior and training exercise can help in developing ways to evaluate rein tension and promote correct use of negative reinforcement.

## Data Availability Statement

The original contributions generated for the study are included in the article/Supplementary Material, further inquiries can be directed to the corresponding author/s.

## Ethics Statement

The animal study was reviewed and approved by the Animal Ethics Board, Uppsala district court, in Uppsala, Sweden.

## Author Contributions

The research objectives and the experimental setup were initiated by ME. Data collection was performed by ME, JY, PB, and AE. Video recordings were made by ME. Data analysis and statistical analysis were performed by ME, AE, and AB. ME wrote the manuscript. All authors contributed to improving the manuscript and refining the experimental design.

## Conflict of Interest

The authors declare that the research was conducted in the absence of any commercial or financial relationships that could be construed as a potential conflict of interest.
